# Characteristics of human - sloth bear (*Melursus ursinus*) encounters and the resulting human casualties in the Kanha-Pench corridor, Madhya Pradesh, India

**DOI:** 10.1371/journal.pone.0176612

**Published:** 2017-04-27

**Authors:** Aniruddha H. Dhamorikar, Prakash Mehta, Harendra Bargali, Kedar Gore

**Affiliations:** 1 The Corbett Foundation, Kanha Division, Madhya Pradesh, India; 2 The Corbett Foundation, Corbett Division, Uttarakhand, India; 3 The Corbett Foundation, Mumbai, Maharashtra, India; Centre for Cellular and Molecular Biology, INDIA

## Abstract

Sloth bears (*Melursus ursinus*) caused the highest number of human deaths between 2001 and 2015 and ranked second compared to other wild animals in causing human casualties in the Kanha-Pench corridor area. We studied the patterns of sloth bear attacks in the region to understand the reasons for conflict. We interviewed 166 victims of sloth bear attacks which occurred between 2004 and 2016 and found that most attacks occurred in forests (81%), with the greatest number of those (42%) occurring during the collection of Non-Timber Forest Produce (NTFP), 15% during the collection of fuelwood and 13% during grazing of livestock. The remainder took place at forest edges or in agricultural fields (19%), most occurring when person(s) were working in fields (7%), defecating (5%), or engaged in construction work (3%). Most victims were between the ages of 31 to 50 (57%) and most (54%) were members of the Gond tribe. The majority of attacks occurred in summer (40%) followed by monsoon (35%) and winter (25%). Forty-four percent of victims were rescued by people, while 43% of the time bears retreated by themselves. In 60% of attacks, a single bear was involved, whereas 25% involved adult females with dependent cubs and the remainder (15%) of the cases involved a pair of bears. We discuss the compensation program for attack victims as well as other governmental programs which can help reduce conflict. Finally, we recommend short-term mitigation measures for forest-dependent communities.

## Introduction

The Sloth bear (*Melursus ursinus* Shaw, 1791, Carnivora: Ursidae: Ursinae), is one of four bear species found in India. It is omnivorous, feeding on social insects such as termites and ants, as well as on fruits such as *Ziziphus mauritiana*, *Ficus benghalensis*, and *Aegle marmelos* [1, 2]. The sloth bear is the only bear having myrmecophagous adaptations, including the absence of the first maxillary incisors, protrusible mobile lips, raised elongated palate, nearly naked mobile snout, slightly curved front claws, long shaggy coat and nostrils which can be closed voluntarily [3]. The sloth bear is endemic to the Indian subcontinent, having geographical distribution across India, Nepal and Sri Lanka. The species has been extirpated from Bangladesh and is reported to be rare in Bhutan [3].

The sloth bear’s range in India extends from the foothills of the Himalayas to the southern tip of the Western Ghats; however, its distribution is non-continuous and fragmented. The central Indian highlands, Western Ghats and the Eastern Ghats are considered to be strongholds of the sloth bear [4, 5]; central India harbors the largest intact habitat and population of bears [6]. The sloth bear is protected under Schedule I of the Wildlife (Protection) Act of 1972, is ranked as Vulnerable on the IUCN Red List, and is listed in Appendix I of Convention on International Trade in Endangered Species of Wild Fauna and Flora (CITES).

There is moderate genetic variation among bears of the Satpuda-Maikal Landscape [7], a part of the central Indian highlands, with corridors between Kanha and Pench Tiger Reserves experiencing active movement of sloth bears, the majority of which is not within any protected area. Of all the carnivores existing in this area, the highest encounter rate recorded was for sloth bears [8].

The corridor between Kanha and Pench Tiger Reserves encompasses an area of approximately 16,000 sq. km. A total of 442 villages exist in the Kanha Pench Corridor [9]. This corridor is actively used by dispersing tigers (*Panthera tigris*) [10] and has been identified as a refuge for other mammals including the leopard (*Panthera pardus*), wild dog (*Cuon alpinus*), gaur (*Bos gaurus*), sambar (*Rusa unicolour*) and chital (*Axis axis*) [11].

Collection of Non-Timber Forest Produce (NTFP) is one of the common income generation activities in this region, with established markets for a number of products such as tendu leaves (*Diospyros melanoxylon*), mahua flowers and seeds (*Madhuca indica*), sal seeds (*Shorea robusta*) and bamboo (*Dendrocalamus strictus*). In 2012, as many as 1.2 million people collected tendu leaves in Madhya Pradesh [12]. Collection of NTFP requires the person to venture several kilometers into the forests, thereby increasing the chance of encounters with sloth bears. Other activities such as cattle grazing and fuelwood collection also increase the risk of sloth bear encounters.

We assessed factors associated with sloth bear attacks by interviewing the victims in two forest divisions (non-protected areas): Balaghat Circle, Seoni Circle, and one protected area, the buffer zone of Kanha Tiger Reserve. We only included attacks that occurred between January 2004 and May 2016 for this analysis. Information collected included the time of the day, season, activity of the victim and the bear, the level of wounds sustained, defense method, frequency of forest visits by the victim, the socio-economic background of the victim, as well as the compensation mechanism of the Forest Department. Here we discuss sloth bear conflict trends in the Kanha-Pench corridor area and the need for mitigation measures.

## Materials and methods

### Ethics statement

The authors confirm that the present study was first submitted to The Corbett Foundation’s senior advisory members (TCF advisory committee) for review and approval before being submitted to a funding agency. Advisory members act as a review committee for wildlife-related research projects as well as for projects requiring participation of local communities through surveys and focused group discussions. This study was deemed acceptable with regard to its ethical approach for interaction with the victims. This study proposal was reviewed and approved for submission to a funding agency for further assessment before being awarded a grant. No third parties, including the members of the funding agency or any government officials were involved in the survey or data analysis process. Verbal confirmation of the victims was sought during the survey and was conducted in the presence of at least two family members of the victim. No written consent or victims’ signatures were obtained as most were unable to read and/or write. People who were able to read and write often refrained from signing any documents due to personal reasons, although they agreed to an oral interview. Therefore, we considered verbal permission to suffice, and the victims’ answers were anonymously recorded. Considering the social constraints of the region, advisory members approved our consent procedure by maintaining participant anonymity.

## Study area

The Kanha-Pench Corridor lies in the southern portion of the Satpuda range called Maikal hills, between N 21°45’15” E 079°30’05” and N 22°24’20” E 080°32’55”, covering an area of approximately 16,000 sq. km [8]. The corridor largely falls in the districts of Balaghat, Seoni and Mandla of Madhya Pradesh and is characterized by small ridges and hills with steep slopes. The region is dominated by moist peninsular sal forests, southern tropical moist mixed and dry mixed deciduous forests and tropical dry teak forests [13]. The Balaghat district has the greatest forest cover in the state (54%), while the Mandla (49%) and Seoni (47%) districts rank fourth and fifth, respectively [14]. The corridor is interspersed with villages, a network of roadways and railway line.

As per human population census estimates (2011), 36% of the residents of these three districts belonged to tribal communities [15, 16, 17]. The major tribal communities included Gond and Baiga, and both were dependent on forests for basic household needs and for at least a part of their income such as the collection of NTFP. The larger portion of the population was comprised of Pawar, Marar, Lodhi, Aahir, and Yadav communities. These communities were largely agrarian and pastoralists but also engaged in the collection of NTFP.

With respect to wildlife-inflicted casualties in Madhya Pradesh, Seoni Circle, Balaghat Circle and Kanha Tiger Reserve ranked 7^th^, 10^th^ and 15^th^, respectively, between 2001 and 2015 [18]. A total of 1,456 incidents of human injury were recorded in these areas by the Forest Department between 2001 and 2015, of which 41% were inflicted by wild pig (*Sus scrofa*), 24% by sloth bear and 22% by jackal (*Canis aureus indicus*). The remaining 13% of animal attacks involved tiger, leopard and langur (*Semnopithecus entellus*). Wildlife-inflicted fatalities numbered 47 in the past 15 years, the majority (*n* = 16) due to sloth bears, followed (in descending order) by wild pig, tiger, and jackal [18].

### Methods

We obtained the addresses of attack victims from the Madhya Pradesh Forest Department. Victims were visited and one-on-one, in-person interviews were conducted through a structured questionnaire. We conducted interviews from February 2016 to May 2016.

#### Victim interviews

We used two standardized questionnaires, one for attacks and another to determine the socio-economic status of the victim. We asked questions that provided information regarding variables associated with sloth bear attacks, such as the date and time of the attack, the activity of the victim during the attack, location of the attack, activity of the bear during the attack, number of bears encountered, the attack pattern of the bear, wounds sustained during the confrontation (an external injury resulting from direct contact with a sloth bear was considered as a wound), as well as the defense method used by the victim (S1 File).

To understand the socio-economic status and the dependency upon forests of the victims, we asked questions pertaining to the average annual income and how that income was derived (e.g., agriculture, manual labor, animal husbandry, NTFP and fuelwood collection). We classified these occupations into a single primary occupation and primary occupation with an alternate source of income (S1 File). Interviewers introduced themselves and informed victims about the current study. Interviews were completely voluntary with the interviewee having the right to terminate the interview at any time. We made an effort to confirm the authenticity of each case during the interview process. Interviews were conducted in Hindi language, in the home of the victim in the presence of two co-authors (AD and PM) and at least two of the victim’s family members.

#### Demographic details

We obtained information related to the local population, gender ratio, and caste for villages in the study area through the Government of India’s population census data for the year 2011 [15, 16, 17]. The caste of each victim was classified into a ‘social group’ based on their distinct customs and ways of living (S2 File). District and sub-district demographic information assisted in separating multiple villages with the same name. In certain cases, the victim’s village was considered a satellite of a larger village so the population of the larger village was used.

#### Data analysis

Our survey contained ‘yes’ and ‘no’ options for wherever specific quantitative data could not be obtained. Raw data were entered into Microsoft Office Excel and analyzed as needed (S3 File). Qualitative data were recorded as ‘one’ for yes and ‘zero’ for no, and a comparison between data sets was made in terms of percentage. Statistical analyses such as the Pearson chi-square test (*χ*^*2*^) and t-test (*t*) were undertaken in the R statistical software (R Core Team 2016; Version 1.0.44) and SPSS (IBM; Statistics Version 24). We used Pearson chi-square test (α = 0.05) to test for significant differences in proportions of groups in our survey data, and pair-wise and independent-sample *t*-test (α = 0.05) to test for significant differences between groups. The data were summarized in terms of mean (*M*) and percent, and the measures of variability recorded in terms of SD and at confidence interval of 95% (95% CI). Bootstrapping was computed with 50,000 iterations at 95% CI.

## Results and discussion

**Fig 1:**
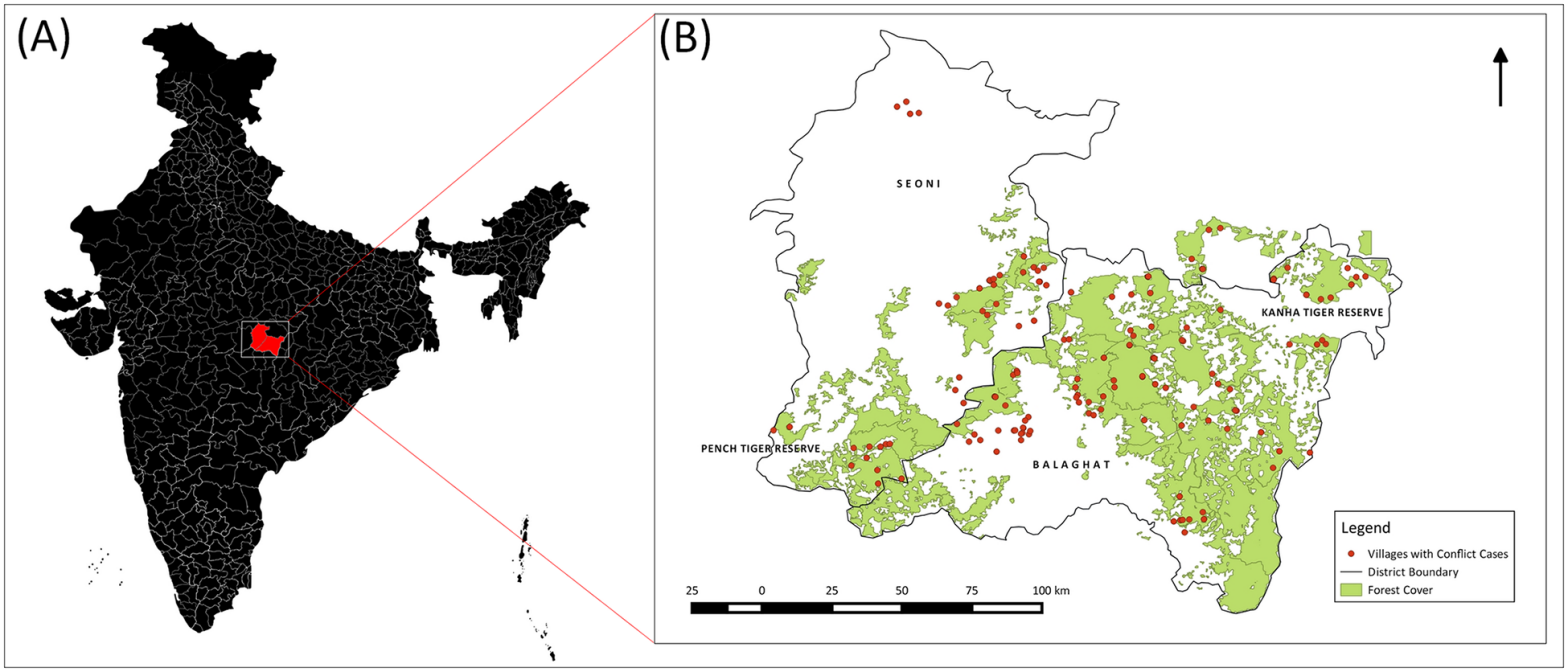
Map showing location of victim’s villages (marked in red) in the Kanha-Pench corridor area in India. Note: (A) Map of India, credited to Anand S, is used with permission to be published under a CC-BY license <https://commons.wikimedia.org/wiki/File:Official_India_Map_with_Districts_2011_Census.svg >. (B) Forest cover map of Kanha-Pench corridor area (in green) showing victim’s villages (marked in red), created under details provided by the Madhya Pradesh Forest Department in public domain <http://www.mpforest.gov.in>. Map not to scale and used for representational purpose only. Map created in QGIS 2.0.

A total of 166 victims (65% of the 255 cases on file) were interviewed from 120 villages in the study area, 77 in the Balaghat district, 37 in the Seoni district, and six in the Mandla district, including the buffer zone of Kanha Tiger Reserve. All 255 interviews could not be conducted because the victim was either unavailable, had moved out of the village, migrated for work or had died from causes unrelated to the attack. Of the total 166 interviews we conducted, 130 were with victims and another 36 with persons accompanying the victim during the attack, or an immediate family member aware of attack details (including parents, spouse and siblings). All villages (Fig 1, S2 File), fell within, or were in close proximity to, the Kanha-Pench corridor area comprising 27% of the total 442 villages identified in the area [9].

### Demographic and socio-economic patterns of victims

#### Age variation

**Fig 2:**
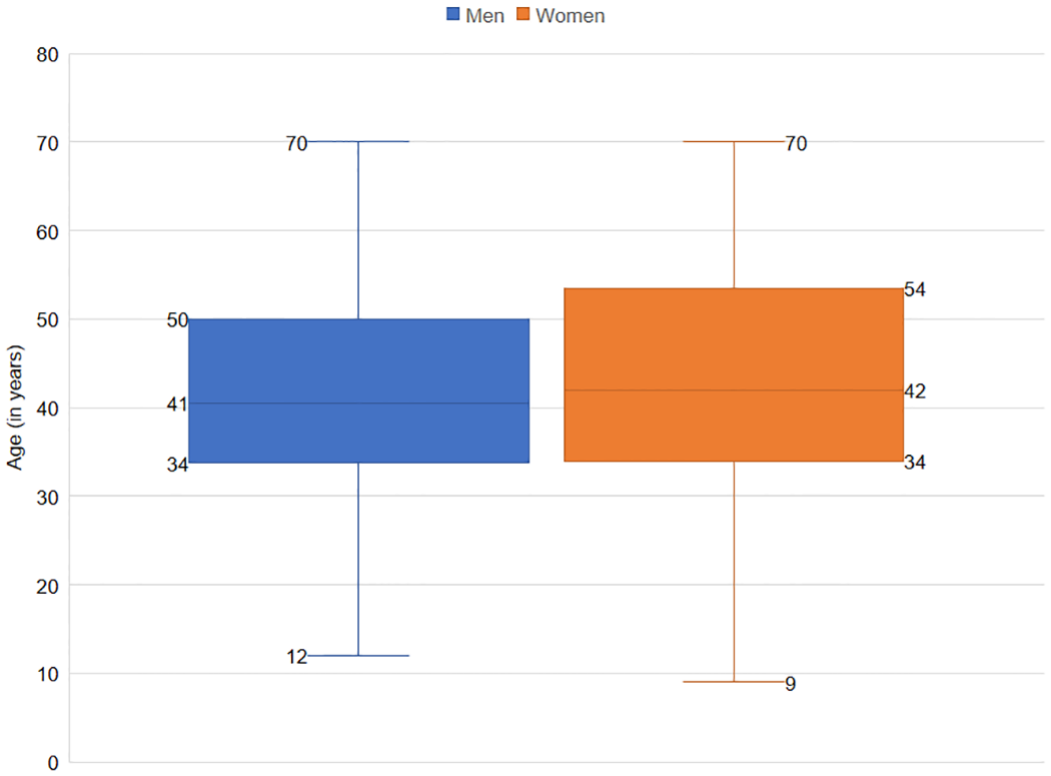
Age variation of sloth bear attack victims, 2004–2005.

Victims’ ages ranged from 9 to 70 years (*M* = 41) at the time of attack. Most victims (29%, *n* = 48) were 31 to 40 years old; 28% (*n* = 47) were 41 to 50 years old; 17% (*n* = 29) were 51 to 60 years old, and 13% (*n* = 21) were 21 to 30 years old (Fig 2). Pearson chi-square test showed a significant difference in the age groups of the victims, *χ*^*2*^(6, *n* = 166) = 49.1, *p* < 0.05.

There was no significant difference between mean age of male (*M* = 41, *SD* = 11.98) and of female (*M* = 41.86, *SD* = 14.85) victims, *t*(164) = -0.35, *p* = 0.72, 95% CI for mean difference -5.29 to 3.70. Bootstrapping at 95% CI showed that the mean age of male victims ranged from 38 to 43 years, and for female victims from 37 to 46 years. Mean difference between ages of male and female victims was between 4.2 to 5.7 years.

We suggest that middle-aged people (37–46 years old) were attacked more because they were more likely to be engaged in outdoor occupations such as collection of forest produce or agriculture around the forested areas and livestock-based activities than the younger (11–30 years old) and older (61–70 years old) age groups.

#### Representation of genders in attack cases

Of the 166 victims interviewed, 75% (*n* = 124) were men and 25% (*n* = 42) were women. There was a significant difference in the proportion of male and female victims, *χ*^*2*^ (1, *n* = 166) = 20.53, p < 0.05. More than half of the female victims (59%, *n* = 25 out of 42) were attacked when they were engaged in NTFP collection, whereas the figure was only 35% (*n* = 44 out of 124) for male victims. For fuelwood collection, 14% (*n* = 6 out of 42) of female victims were attacked as compared to 15% (*n* = 19 out of 124) of male, and 7% (*n* = 3) of female victims were attacked during defecation as compared to 5% (*n* = 6) for male victims. We found no significant difference between the activities of male (*M* = 12.4, *SD* = 12.8) and female (*M* = 4.2, *SD* = 7.5) victims, *t*(18) = 1.75, *p* = 0.097, 95% CI for mean difference -1.65 to 18.05.

A majority of the cases involving female victims occurred during summer (74%, *n* = 31) whereas attacks on male victims were more frequent during the monsoon season (44%, *n* = 54). We found no significant differences between attacks on male (*M* = 41.33, *SD* = 11.01) and female (*M* = 14, *SD* = 14.8) victims across three seasons, viz. summer, monsoon, and winter, *t*(4) = 2.56, *p* = 0.062, 95% CI for mean difference -2.24 to 56.90. Attacks by wild animals in Marwahi Forest Division (Chhattisgarh) have been noted to occur independent of gender [19].

#### Social groups

**Fig 3:**
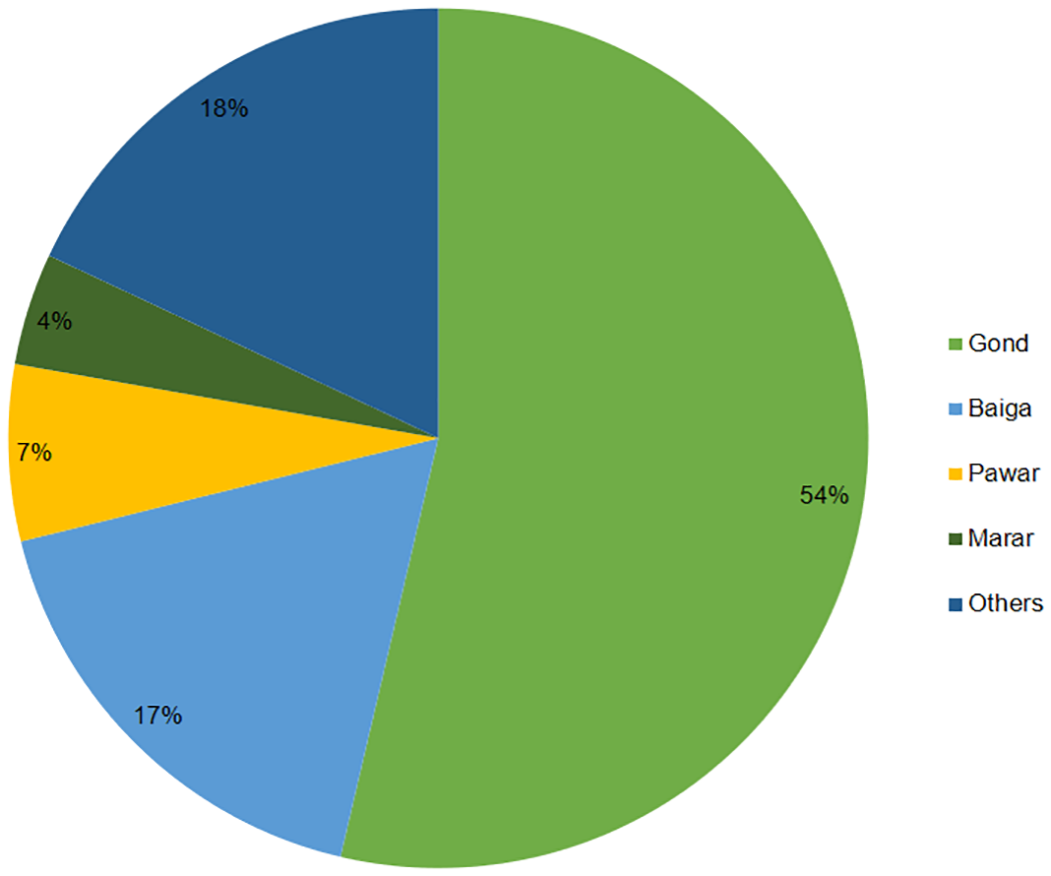
Composition of sloth bear attack victims as per social groups.

We classified victims by social groups based on their caste. We considered social groups as an important variable because social groups strongly influenced an individual’s lifestyle, livelihood and occupation. We identified social groups (*n* = 21) through interviews. The Gond, Baiga, Pawar and Marar caste accounted for the majority (82%, *n* = 136) of attack cases. More than half of the victims belonged to the Gond caste (54%, *n* = 89) followed by 17% (*n* = 29) belonging to Baiga caste, seven percent (*n* = 11) belonged to Pawar caste and four percent (*n* = 7) to Marar caste. The remainder belonged to 17 communities amounting to 18% (*n* = 30) of the total cases recorded (Fig 3). We found a significant difference in the distribution of attacks among different castes, *χ*^*2*^(20, *n* = 166) = 149.74, *p* < 0.05.

The proportion of the tribal population (including Baiga and Gond as major tribal communities) of the three districts (i.e., Balaghat, Seoni, and Mandla) amounted to 36% of the total population, whereas that of the 120 villages to which the victims belonged was 38% [15, 16, 17]. Our study documented that 71% of victims belonged to Baiga and Gond tribes. We found a significant difference in the attack cases involving tribal and non-tribal communities (*χ*^*2*^(1, *n* = 166) = 11.03, *p* < 0.05), suggesting that tribal communities represented more in conflict cases than non-tribal communities.

#### Occupation and income generation

Agriculture and manual labor represented 13% (*n* = 21) and 26% (*n* = 43) of occupations undertaken by the victims, respectively, followed by fishery, carpentry, bamboo harvesting, and business (four percent, *n* = 7).

On average, individual income generated from agriculture amounted to $223 USD (INR 14,876) per year based on responses from 113 respondents while labor amounted to $143 USD (INR 9,539) per year based on responses from 140 respondents. Income of victims involved in fishery, carpentry, shop-keeping and smithery amounted to $125 USD (INR 8,500) per year.

We performed a one-sample t-test to determine whether the mean annual income per victim was different than the Madhya Pradesh state’s average annual income for a household ($346 USD, INR 23,112.7) [20]. Average annual income of victims (*M* = 10130, *SD* = 6125.33) was significantly less than the average per household income for the state, *t*(6) = -5.607, *p* = 0.01, 95% CI for mean difference -18646.97 to -7316.99. Average annual income of victims ($149 USD, INR 10,130), was less than the global average income per person per year ($693 USD, INR 47,200) [21].

### Seasonality and temporal variations of encounters

#### Yearly, monthly and seasonal variation

Of the 255 attacks between 2004 and 2016, 20 were recorded per year (*n* = 31 in 2006, *n* = 9 in 2015). Distribution of attacks was not significantly different from expected over the period of 13 years, *χ*^*2*^(12, *n* = 255) = 18.28, *p* = 0.1. Most attacks occurred in May (19%, *n* = 31), followed by March (12%, *n* = 20) and August (11%, *n* = 19); however, attack frequency did not vary significantly between months, *χ*^*2*^(11, *n* = 166) = 14.91, *p* = 0.18.

**Fig 4:**
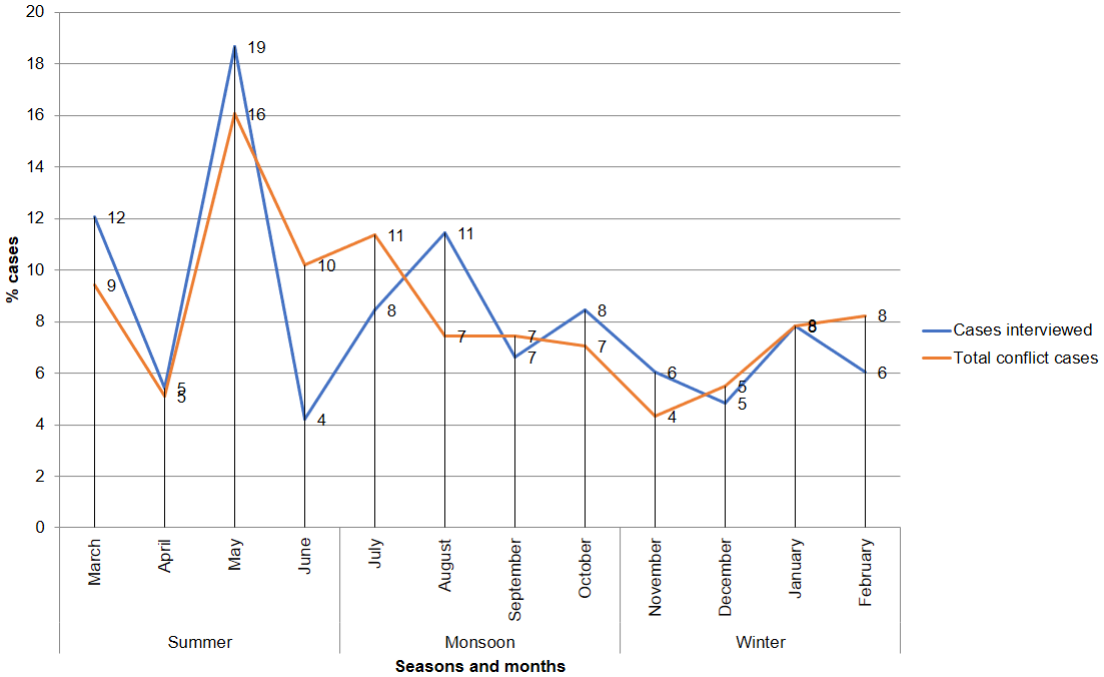
Seasonal and monthly variation of attack cases for victims interviewed (*n* = 166) and cases on file (*n* = 255) in percent.

Seasonally, 40% (*n* = 67) of the attacks took place during summer, 35% (*n* = 58) during monsoon and 25% (*n* = 41) in winter (Fig 4). We corroborated these data with additional information obtained from the Forest Department for a period of 13 years. The seasonal pattern for 255 cases showed a similar trend of greater attack rates during summer than monsoon followed by winter. We did not find significant differences in seasonal attack patterns for either of the sample in our study (*χ*^*2*^(2, *n* = 166) = 3.3, *p* = 0.19) or the cases on file (*χ*^*2*^(2, *n* = 255) = 4.3, *p* = 0.11).

We compared the seasons to examine the difference in cases using an independent sample t-test. The test was performed for three combinations (summer-winter, summer-monsoon and winter-monsoon). Attacks in summer (*M* = 16.75, *SD* = 11.08) were more frequent than in monsoon (*M* = 14.5, *SD* = 3.31) and winter (*M* = 10.25, *SD* = 2.06). There was no significant difference between the number of cases within the three combinations (Table 1).

**Table 1:** Results of t-test for comparison of sloth bear encounters between two seasons.

**Group A**	**Summer**			**Winter**			**Mean Difference**	**95% CI for Mean Difference**	***t***	***df***	***p***
	***M***	***SD***	***n***	***M***	***SD***	***n***					
Conflict cases	16.75	11.08	67	10.25	2.06	41	6.5	-7.29, 20.29	1.153	6	0.29
**Group B**	**Summer**			**Monsoon**			**Mean Difference**	**95% CI for Mean Difference**	***t***	***df***	***p***
	***M***	***SD***	***n***	***M***	***SD***	***n***					
Conflict cases	16.75	11.08	67	14.5	3.31	58	2.25	-11.90, 16.40	0.389	6	0.71
**Group C**	**Winter**			**Monsoon**			**Mean Difference**	**95% CI for Mean Difference**	***t***	***df***	***p***
	***M***	***SD***	***n***	***M***	***SD***	***n***					
Conflict cases	10.25	2.06	41	14.5	3.31	58	-4.25	-9.02, 0.52	-2.177	6	0.07

We found that an increase in attack frequency during the months of March, May and August (Fig 4) was correlated to an increase in forest visits for the collection of NTFP. The month of March is when the collection of mahua flowers begins and May is when tendu leaf collection season begins. Towards the end of July and August, victims were usually engaged in wild mushroom harvest, a product which was consumed directly while the surplus was sold in an open market. Attacks during the remainder of the year, especially in winter, were correlated with frequency of visits to forests for fuelwood collection (24%, *n* = 10), grazing (22%, *n* = 9) and NTFP collection (17%, *n* = 7).

The majority of sloth bears attacks in the neighboring state of Chhattisgarh occurred during the monsoon season [22]. Chhattisgarh is geographically similar to the Kanha-Pench corridor area, albeit with a different social composition. Attacks by Asiatic black bear (*Ursus thibetanus*) in Dachigam National Park (Kashmir) were mostly reported during May to November [23], while the encounters with Asiatic black bear and Himalayan brown bear (*Ursus arctos isabellinus*) in the Great Himalayan National Park Conservation Area (Himachal Pradesh) took place when villagers ventured into forests for fuelwood, fodder, medicinal plants or to graze livestock, irrespective of the season [24]. Attacks by bears during late August to September were recorded in the Sichuan Province in southwestern China where the Asiatic black bears confronted wild mushroom harvesters [25].

#### Attack timing

**Fig 5:**
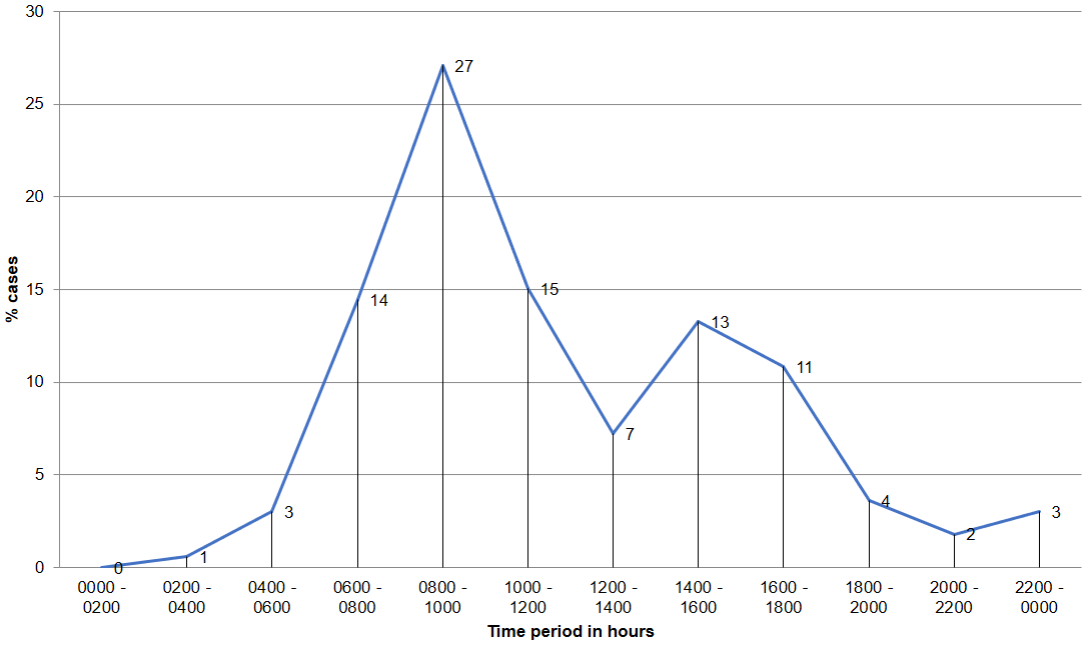
Conflict cases against a 24-hour timescale in percent.

We divided each day into twelve two-hour periods to analyze patterns in timing of attacks. Most cases (27%, *n* = 45) occurred between 0800 and 1000 hrs., followed by 15% (*n* = 25) between 1000 and 1200 hrs., 14% (*n* = 24) between 0600 and 0800 hrs. and 13% (*n* = 22) between 1600 and 1800 hrs. Four percent (*n* = 6) of the encounters were recorded during early morning hours (0200–0600) and five percent (*n* = 8) of the cases were recorded between 2000 and 0000 hrs. (Fig 5). The difference between attack timing was significant, *χ*^*2*^(11, *n* = 166) = 68.28, *p* < 0.05. This finding may be related to patterns of forest visitations by the local people in the study area and/or the variation in sloth bear activity.

On comparing our findings with studies from North Bilaspur (Chhattisgarh), where most attacks (45%) were reported during the early morning hours (0400–0800 hrs.) [22], we found that most attacks in our study occurred between 0800 and 1000 hrs. (27%, *n* = 45), whereas 14% (*n* = 24) occurred between 0600 and 0800 hrs. We recorded that 64% (*n* = 106) of the victims visited forests during morning and returned by noon, and 36% (*n* = 60) visited forests during morning as well as early evening hours and returned before sunset. In terms of the duration of visits to the forests, 37% (*n* = 61) visited for two to three hours, 35% (*n* = 58) for more than three hours, and 28% (*n* = 47) for less than two hours.

### Spatial variations of the encounters

#### Victim activity during the attacks

**Fig 6:**
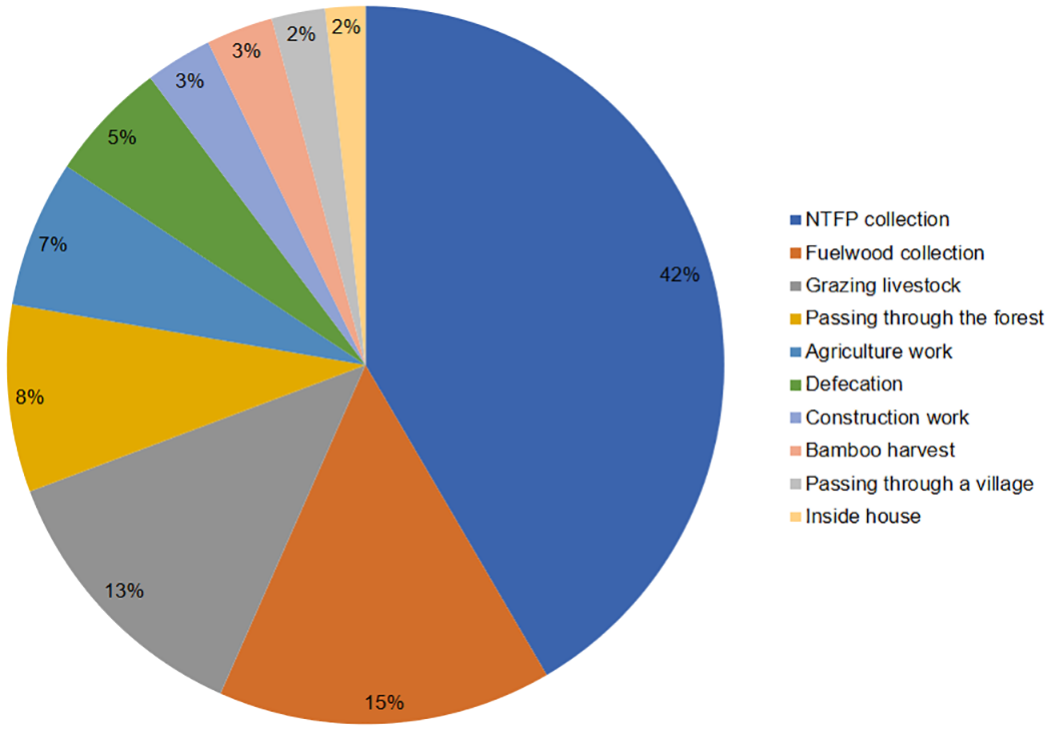
Activities of the victims during the attack in percent.

Most victims (42%, *n* = 69) were engaged in NTFP collection during attacks while 15% (*n* = 25) were attacked during fuelwood collection and 13% (*n* = 21) during livestock grazing in forests. Moving through the forest for an errand accounted for eight percent (*n* = 14) of attacks, while passing through a village accounted for two percent (*n* = 4) of attacks. Open-area defecation in agricultural fields adjacent to forests resulted in five percent (*n* = 9) of the attacks, whereas working in agricultural fields in seven percent (*n* = 11); construction activity and bamboo harvesting each accounted for three percent (*n* = 5) of attacks. In two percent (*n* = 3) of the cases we studied, the sloth bear had reportedly entered a house in search of food (Fig 6).

The proportion of individuals engaged in certain activities varied significantly from expected values, *χ*^*2*^(9, *n* = 166) = 67.5, *p* < 0.05. Attacks during agricultural work, construction activity, defecation, and livestock grazing along the edge of forest areas signified that the sloth bears frequented areas with multiple land uses, especially closer to village edges. In North Bilaspur (Chhattisgarh), the presence of sloth bears close to human habitations was found to indicate the use of degraded habitats by the bears [26].

#### Attack locations

We classified attack locations as forests, agricultural fields, or villages. Most encounters took place in forests (81%, *n* = 134), with 12% (*n* = 20) and seven percent (*n* = 12) occurring in agricultural fields, often at the forest’s edge, and within village boundaries, respectively. Most encounters (39%, *n* = 65) occurred within 1 km of the victim’s home, while 29% (*n* = 48) and 17% (*n* = 29) occurred within 3 km and within 5 km, respectively. A majority of the confrontations in the forest occurred within 3 km of the victim’s home (62%, *n* = 84 out of 134) whereas and 19% (*n* = 26 out of 134) within 5 km of the victim’s home.

#### Attacks during forest-based activities

Of the total attacks that occurred in forests (81%, *n* = 134), half of the encounters took place during NTFP collection (51%, *n* = 69), 19% (*n* = 25) during fuelwood collection, 16% (*n* = 21) during livestock grazing, and the remaining 14% (*n* = 19) during bamboo harvest and while passing through the forest. Attack frequency was not significantly different between forest (*M* = 26.8, *SD* = 24.78) and in non-forest settings (*M* = 6.4, *SD* = 3.43), *t*(8) = 1.823, *p* = 0.106, 95% CI for mean difference -5.40 to 46.20.

In case of NTFP collection, 32% (*n* = 22) attacks occurred during tendu leaf collection followed by 25% (*n* = 17) during wild mushroom collection, 20% (*n* = 14) during mahua flowers collection, 19% (*n* = 13) during maulian leaf (*Bauhinia vahlii*) collection and four percent (*n* = 3) during collection of other NTFP such as chhind leaves (*Phoenix acaulis*), amla fruits (*Phyllanthus emblica*) and char fruits (*Buchanania lanzan*). Sloth bear sightings have also been noted during the collection of honey from combs of the giant honey bee (*Apis dorsata*) however no attacks have been reported. We found that attacks during NTFP collection were more likely because the collectors entered forests in large numbers and engaged in the gathering activity silently and separately, increasing their chances of sudden encounters with sloth bears.

### Activity and behavioral patterns during attacks

#### Number of bears involved

We grouped the number of bears involved in these attacks into two categories, single and multiple. Most victims 60% (*n* = 99) were attacked by a single bear and 40% (*n* = 67) involved two or more bears, although no cases involved multiple bears attacking at the same time.

**Fig 7:**
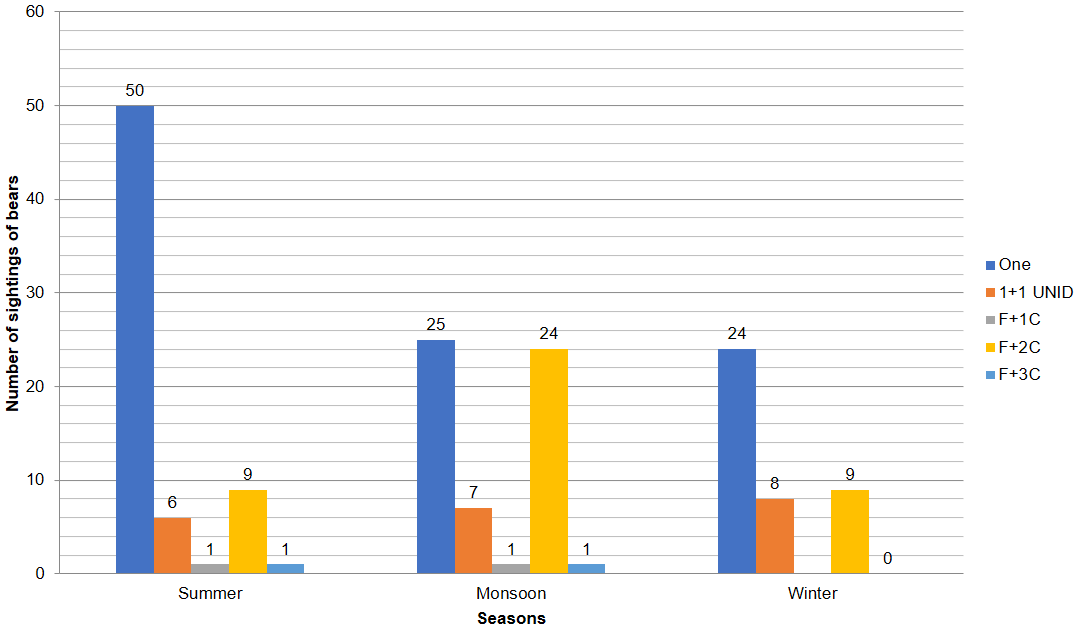
Number of bear sightings during summer, monsoon, and winter. 1+1 UNID = two bears of unknown gender and age; F+1C = 1 female with 1 cub; F+2C = 1 Female with 2 cubs; F+3C = 1 Female with 3 cubs.

A group of two or more bears were further categorized into two (unidentified), two (one female with one cub), three (one female with two cubs), and four (one female with three cubs). The number of bears reported by victims, when analyzed by season, showed the following trend: most cases involved a single sloth bear, of which 75% (*n* = 50 of the total 99 cases) occurred during summer and most cases involving 3 bears occurred during monsoon (57%, *n* = 24 of the total 42 cases). Attacks involving two bears occurred uniformly throughout the seasons (Fig 7).

#### Bear activity during attacks

**Fig 8:**
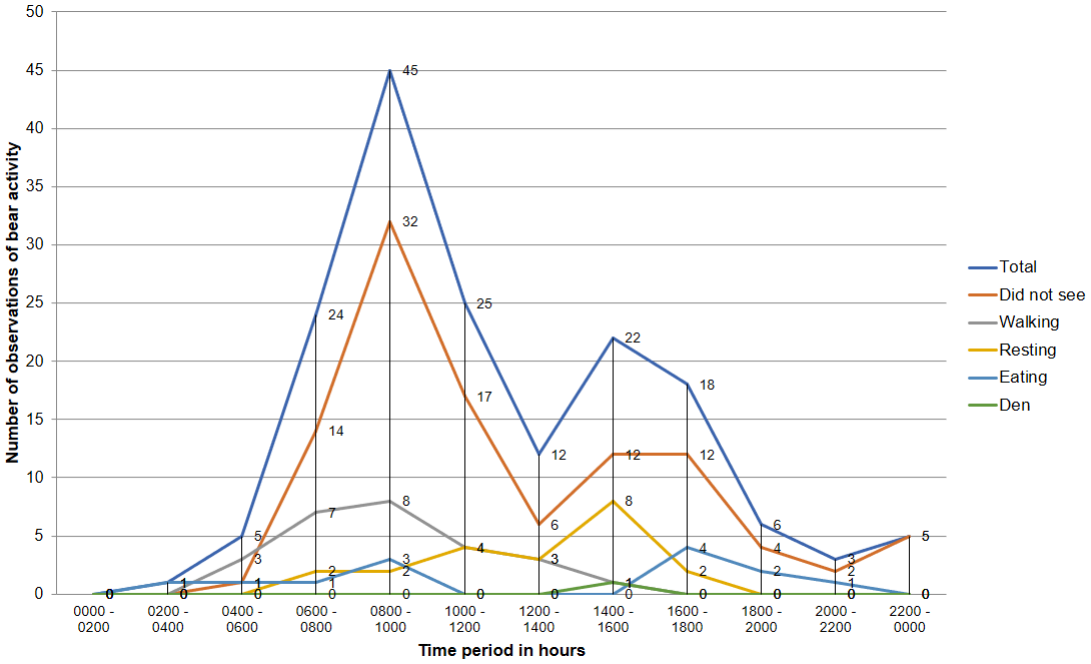
Activity of the sloth bear prior to the attack against a 24-hour timescale.

A total of 63% (*n* = 105) of victims did not see the bear before it attacked, 16% (*n* = 26) saw the bear walking and observed it cross their path, 13% (*n* = 21) said the bear was resting in the bush (*Lantana camara* shrubs) while eight percent (*n* = 13) saw the bear feeding (two respondents independently said they saw the bear eating mahua flowers and ber fruits (*Ziziphus mauritiana*)), and one observed the bear come out of a den (Fig 8).

#### Method of attack

We found that in 49% (*n* = 82) of cases the bear approached the victim from the front and in 43% (*n* = 72) from behind, while four percent and three percent reported that the bear charged from behind a bush or rocks, respectively. We could not confirm the direction of encounter of three victims who succumbed to the injuries from the bear attack. In 67% (*n* = 111) of incidents, the bear stood up and in 21% (*n* = 35) incidents it vocalized after charging towards the victim. On contact, the bear knocked down 36% (*n* = 59) of the victims whereas the remainder reported that they fell on their own. Once the victim was on the ground, the bear used claws (86%, *n* = 142) as well as teeth (72%, *n* = 119) to attack.

### Mode of wounds and defense mechanism adopted by victims

#### Wounds sustained and victim response during the attack

We classified wounds sustained from confrontations as single, double, or multiple wounds. Majority of victims received a single wound (42%, *n* = 69), while 31% (*n* = 52) and 25% (*n* = 42) sustained double and multiple wounds, respectively. Two percent (*n* = 3) of the victims died of multiple wounds. Most wounds were received on the legs (32%, *n* = 102), while 27% (*n* = 87) also received injuries to the hand, followed by 16% (*n* = 51) on the head and back each, and eight percent (*n* = 26) on the stomach. Three victims were also injured on the neck and chest and one received secondary wounds by falling. No fractures were recorded. Instances of wounds to legs and hands were probably relatively great because victims tried to defend themselves after falling. Injuries to stomach and back were reported to have been caused by claws, and those to the neck by teeth. There was no significant difference in the nature of wounds between male (*M* = 31, *SD* = 21.9) and female (*M* = 10.5, *SD* = 6.4) victims, *t*(6) = 1.797, *p* = 0.12, 95% CI for mean difference -7.40 to 48.40.

#### Victims’ group size

In most cases (41%, *n* = 68), victims were alone, but 22% (*n* = 36) were in a pair, 20% (*n* = 34) were in a company of three, and 17% (*n* = 28) were with more than three people. There was a significant difference in victim group size, *χ*^*2*^(3, *n* = 166) = 12.89, *p* < 0.05. Female victims were more likely to work in groups of more than three than alone, which was the opposite case for male victims who often worked alone. Of the female victims attacked, 33% (*n* = 14) were in a group of more than three whereas 11% (*n* = 14) of male victims were in groups of three or more. In contrast, 26% (*n* = 11) of female victims were working alone whereas 46% (*n* = 57) of male victims were working individually during attacks.

We compared victim activity with group size and found that people engaged in a forest-dependent activity, namely NTFP and fuelwood collection, often went in groups of three or more. However, these individuals often split into smaller groups upon reaching the forests, likely increasing their chances of confronting bears alone. Male victims ventured into forests alone or in pairs during the collection of NTFP, which may account for the increased prevalence of attacks on males. When working in fields or at the forest edge (e.g., when defecating or grazing livestock), victims were either alone or in groups of two (Table 2).

**Table 2:** Activity and group sizes of victims.

Activity of the victim	Group Size				
	Single	Double	Three	>Three	Total cases
*NTFP collection*	17	18	15	19	69
*Fuelwood collection*	9	4	6	6	25
*Grazing livestock*	15	4	2	0	21
*Walking in forest*	7	3	4	0	14
*Agriculture work*	8	2	1	0	11
*Defecation*	8	1	0	0	9
*Construction work*	2	1	0	2	5
*Bamboo harvest*	0	1	3	1	5
*Walking in village*	3	1	0	0	4
*Resting in house*	3	0	0	0	3
Total	72	35	31	28	166

#### Defense method

We found that 44% (*n* = 73) of victims were saved by people who came to rescue, yet, in 43% (*n* = 71) of the attacks, the bear retreated before help arrived. In seven percent (*n* = 12) of the attacks, animals accompanying the victim(s) (e.g. cattle, dog) intervened. In such cases, the bear often went after the animal, providing the victim an opportunity to escape. In six percent (*n* = 10) of attacks, the victim used an axe, stone, or stick in self-defense. Victims mostly received single (42%, *n* = 69) or double (32%, *n* = 53) wounds in most cases irrespective of method(s) of self-defense.

### Expected vs actual compensation

The Government of Madhya Pradesh provides compensation for citizens wounded by wild animals [18]. In this study, we found that the minimum compensation for those who were injured in sloth bear attacks was $3 USD (INR 200) and the maximum $449 USD (INR 30,000) (*M* = $73 USD, INR 4,862). Compensation for victims who perished ranged from $1,497 USD (INR 1,00,000) to $2,245 USD (INR 1,50,000). The minimum time to receive compensation was the same day and the maximum was 24 months. Forty percent (*n* = 67) received compensation on the same day, nine percent (*n* = 15) two months after the attack and six percent (*n* = 9) one month after the attack. The remainder 17% (*n* = 28) received compensation anywhere between two days to 24 months and 28% (*n* = 47) did not disclose the compensation amount.

There was a significant difference in the mean compensation received (*M* = 5106.7, *SD* = 16824.2) and expected (*M* = 18389.2, *SD* = 18828.1), *t*(275) = -6.08, *p* < 0.05, 95% CI for mean difference -17582.51 to -8982.51. Bootstrapping at 95% CI showed that mean compensation received ranged from $38 USD (INR 2,641) to $126 USD (INR 8,591), and expected compensation from $60 USD (INR 4,112) to $395 USD (INR 26,897). Mean difference at 95% CI between received and expected compensation was between $137 USD (INR 8,886.42) and $267 USD (INR 17,331.78).

Most victims (96%, *n* = 114) received compensation under $224 USD (INR 15,000), of which 86% (*n* = 98 out of 114) received under $75 USD (INR 5,000) irrespective of wound severity and gender. In terms of medical expenses, 48% (*n* = 77) of victims received compensation for treatment through the Forest Department whereas 52% (*n* = 86) bore the cost themselves. The expected compensation was 21% times more than the received compensation. In cases which were immediately forwarded to the Forest Department, the department took the responsibility of taking the victim to the hospital and a certain amount (in this study, $7.5 USD, INR 500) was paid to victims upfront to cover basic medical costs.

## Conclusions

Understanding conflict between humans and wild animals is important because it can promote conservation efforts for animal species, especially in the case of large carnivores such as felids, canids, and ursids. Nonetheless, casualties resulting from such interactions undermine conservation efforts and encourage retaliatory killings of wild animals [26, 27, 28]. A lack of understanding can impact landscape-level conservation initiatives which require participation of local communities.

The Kanha-Pench corridor area is a mosaic of land-use patterns including two important Tiger Reserves, Kanha and Pench, reserved forests, farmlands, villages, two major district headquarters of Balaghat and Seoni as well as a network of roads and a railway line. There are several large and small scale mines of Copper, Manganese and Coal [29]. The region also supports relatively great biodiversity and is considered one of the most important refuges for tigers in the central Indian landscape [10]. Even non-protected areas in this region support resident tiger, leopard, gaur, and sloth bear populations [8].

Through this study, we provide a better understanding of temporal and spatial patterns of sloth bear attacks, demography of the vulnerable population, reasons of conflict, and existing mitigation measures. We conclude that most attacks occurred when people ventured into forest tracts for the extraction of NTFP and fuelwood, both crucial livelihood sustaining activities for the people of this region. Furthermore, 71% of the attack victims belonged to tribal communities signifying that tribal communities were attacked more often because a significant portion of their livelihood depended upon the habitat shared with the sloth bears. On average, victims lived well below the poverty level based on their primary occupations which likely intensified their dependency on forest for household purposes (such as procuring fuelwood), and as an alternate livelihood option (such as NTFP collection) for income generation.

We conclude that attacks on males were more frequent than on females because men ventured into forests more often alone than in groups. Both male (35%, *n* = 44 out of 124) and female (60%, *n* = 24 out of 42) victims were attacked more while involved in NTFP collection. We found that middle-aged people between 37 and 46 years of age numbered more in terms of encounters with sloth bears because they engaged more in forest-based activities.

Attacks during fuelwood collection ranked second in frequency (15%), although 96% depended on forests for fuelwood collection. Grazing livestock ranked third in terms of activity during attacks (13%), although 77% of victims own large animals and graze livestock in the forest. Attacks during agricultural work and defecation (12%) occurred at the forest edge where these activities typically occur. We conclude that bear attacks of this nature, as well as those which took place while passing through a forest or a village, could have been avoided by using devices which alert the bears of human presence. Use of sounds to avoid sudden confrontations and avoidance of travelling alone during nighttime hours, or by travelling in groups, may reduce conflict.

It is critical to have a mitigation plan in place to minimize future confrontations. In terms of short-term mitigation measures, we propose training and capacity building of the Forest Department staff, as well as the villagers, as to how they can avoid sudden confrontations, what they should do in case of a confrontation, what to do as post-confrontation measures in terms of giving first aid and quick medical services, and educate the community regarding the existing compensation program.

We found that sloth bear attacks were unintentional, unlike most cases involving large mammals such as tigers, leopards, wild pigs, and elephants (*Elephas maximus*). In most instances, humans venturing into forests for livelihood purposes and surprising a bear was the most common cause of attack. It is important to address issues of conflict with sloth bears without alienating people from their livelihood; however, reducing dependency on forests in a sustainable manner by providing alternate income-generating options will ultimately reduce conflict.

The process of NTFP collection for income generation is managed by government, enabling local communities to legally engage in the activity. In terms of tendu leaf collection, the Forest Department provides insurance to collectors [12] in case of injury or death during collection. Tendu leaf and bamboo harvesters are also provided a bonus, a share of profits, generally paid one or two years later [30]. Similar mechanisms of insurance and bonus payments may be initiated for commercial NTFP products, which will empower local communities to gain access to healthcare without monetary constraints. In addition, because NTFP collection is a legal activity, obligatory training workshops for collectors at the community, village, and district level can be developed to prepare participants in adopting preventive methods aimed to avoid confrontations with sloth bears and other potentially dangerous wild animals.

Government programs such as ‘Pradhan Mantri Ujjwala Yojana’ under which people living below the poverty line receive a Liquefied Petroleum Gas (LPG) connection free of charge [31] was launched to reduce health hazards associated with burning biomass. This program could be promoted in this region, thus resulting in a reduction of fuelwood dependency which may also aid in reducing conflict. In India, fuelwood accounts for about 60% of total fuel in rural areas [32]. Programs such as ‘Swachh Bharat Mission’ [33] ensures provision of a toilet for every household in rural India through the Village Council (Gram Panchayat) with an aim to increase hygiene and sanitation in rural areas. This program may also assist in reducing conflict with wildlife by discouraging people from open-area defecation. Grazing of livestock in forests was also a factor that resulted in conflict. Encouragement of stall feeding (i.e. the use of feedlots) through incentives such as providing better yielding variety of livestock and assisting subsistence animal owners in growing fodder crops, will help reduce human and livestock exposure to wild animals. Such actions may also assist in reducing anthropogenic pressure on forest regeneration.

We found that victims unfamiliar with the process of applying for compensation lacked timely care due to insufficient funds. The process of receiving compensation is predetermined and streamlined by the Government of Madhya Pradesh under the Madhya Pradesh Guarantee of Public Service Delivery Act 2010 [34]. Incidents on land managed by the Revenue Department are forwarded to district-level government officials (Tehsildar/ Additional Tehsildar/ Naib Tehsildar) and the typical timeline provided for compensation is 30 days. When an attack occurs on land managed by the Forest Department (Territorial Division and Protected Area), details of the attack are first forwarded to the Range Officer and the timeline for providing compensation is set to seven days for injuries and three days for death caused by wild animals [34]. Generating awareness about the existing compensation program will assist victims in accessing monetary support from the government.

With an increasing human population in India, the pressure on natural ecosystems is increasing [35] and sloth bear habitat is shrinking and becoming more fragmented [3]. In comparison with the outreach of studies on human—wildlife conflict involving large mammals such as tigers, leopards and elephants, focus on human—sloth bear conflict has been considerably less [28], perhaps undermining stronger conservation measures for this species.

Understanding the characteristics of conflict with sloth bears is one way of developing on the ground models which can help to improve conflict mitigation by creating strategies which require the integration and implementation of government programs and active participation of local people, along with the participation of non-governmental and related governmental organizations. This multi-pronged approach of conflict mitigation and reduced anthropogenic pressure on shared habitat is especially crucial for sloth bear conservation because they inhabit human-dominated landscape and exist in relatively large numbers outside protected areas such as the Kanha-Pench corridor.

## Supporting information

S1 File Data collection format for conflict and socio-economic survey. (XLSX)

S2 File Demographic characteristics of respondents. (XLSX)

S3 File Descriptive statistics of variables. (XLSX)
